# A general prediction model for compound-protein interactions based on deep learning

**DOI:** 10.3389/fphar.2024.1465890

**Published:** 2024-09-04

**Authors:** Wei Ji, Shengnan She, Chunxue Qiao, Qiuqi Feng, Mengjie Rui, Ximing Xu, Chunlai Feng

**Affiliations:** ^1^ School of Pharmacy, Jiangsu University, Zhenjiang, China; ^2^ School of Medicine, Jiangsu University, Zhenjiang, China

**Keywords:** compound-protein interaction, deep learning-based prediction model, unbiased large-scale negative dataset, generalization capability, traditional Chinese medicine

## Abstract

**Background:**

The identification of compound-protein interactions (CPIs) is crucial for drug discovery and understanding mechanisms of action. Accurate CPI prediction can elucidate drug-target-disease interactions, aiding in the discovery of candidate compounds and effective synergistic drugs, particularly from traditional Chinese medicine (TCM). Existing *in silico* methods face challenges in prediction accuracy and generalization due to compound and target diversity and the lack of largescale interaction datasets and negative datasets for model learning.

**Methods:**

To address these issues, we developed a computational model for CPI prediction by integrating the constructed large-scale bioactivity benchmark dataset with a deep learning (DL) algorithm. To verify the accuracy of our CPI model, we applied it to predict the targets of compounds in TCM. An herb pair of *Astragalus membranaceus* and *Hedyotis diffusaas* was used as a model, and the active compounds in this herb pair were collected from various public databases and the literature. The complete targets of these active compounds were predicted by the CPI model, resulting in an expanded target dataset. This dataset was next used for the prediction of synergistic antitumor compound combinations. The predicted multi-compound combinations were subsequently examined through *in vitro* cellular experiments.

**Results:**

Our CPI model demonstrated superior performance over other machine learning models, achieving an area under the Receiver Operating Characteristic curve (AUROC) of 0.98, an area under the precision-recall curve (AUPR) of 0.98, and an accuracy (ACC) of 93.31% on the test set. The model’s generalization capability and applicability were further confirmed using external databases. Utilizing this model, we predicted the targets of compounds in the herb pair of Astragalus membranaceus and Hedyotis diffusaas, yielding an expanded target dataset. Then, we integrated this expanded target dataset to predict effective drug combinations using our drug synergy prediction model DeepMDS. Experimental assay on breast cancer cell line MDA-MB-231 proved the efficacy of the best predicted multi-compound combinations: Combination I (Epicatechin, Ursolic acid, Quercetin, Aesculetin and Astragaloside IV) exhibited a half-maximal inhibitory concentration (IC_50_) value of 19.41 μM, and a combination index (CI) value of 0.682; and Combination II (Epicatechin, Ursolic acid, Quercetin, Vanillic acid and Astragaloside IV) displayed a IC_50_ value of 23.83 μM and a CI value of 0.805. These results validated the ability of our model to make accurate predictions for novel CPI data outside the training dataset and evaluated the reliability of the predictions, showing good applicability potential in drug discovery and in the elucidation of the bioactive compounds in TCM.

**Conclusion:**

Our CPI prediction model can serve as a useful tool for accurately identifying potential CPI for a wide range of proteins, and is expected to facilitate drug research, repurposing and support the understanding of TCM.

## 1 Introduction

Accurate identification of compound-protein interactions (CPIs) is a crucial foundation of drug discovery as it can accelerate the hit identification and is also helpful for understanding the underlying mechanism of action of a drug (Lounkine et al., 2012; Oprea and Mestres, 2012). Accurate and comprehensive identification of CPIs enables the interpretation of intricate interactions among drugs, targets, and diseases, thereby facilitating a profound understanding of the therapeutic effects of individual drugs as well as multi-drug regimens.

The identification of CPIs is typically confirmed through wet-laboratory experimentation. However, such experimental validation, including high-throughput screening and other bioassays, remains time- and cost-consuming, hinging its success upon the vast expanse of compound space (Hay et al., 2014; Mak and Pichika, 2019). In contrast, computational methods have been harnessed for CPI prediction. Traditional computational methods mainly include approaches like ligand-based and structure-based virtual screening, and 3D shape matching. Nevertheless, poor prediction performance has been documented, attributed to a scarcity or absence of known ligand information and protein structures. Instead, regarding structure-free methods, machine learning-based models have been developed to predict CPI. Common machine learning algorithms include support vector machine (SVM), K-nearest neighbor (KNN), random forest (RF) and extreme gradient boosting (XGBoost) (Chen et al., 2016; Bagherian et al., 2021; Yu et al., 2022). For example, with the MACCS substructure fingerprints of compounds and physicochemical properties of proteins, an SVM model achieved good prediction performance for four datasets, including enzymes, ion channels, G protein-coupled receptors (GPCRs) and nuclear receptors (Cao et al., 2012). Additionally, regarding these four datasets, another RF model named LRF-DTI obtained a good prediction accuracy, which used the pseudo-position specific scoring matrix (PsePSSM) and FP2 molecular fingerprint to extract interaction features (Shi et al., 2019). However, most traditional machine learning approaches require training models for each protein separately, limiting the model’s applicability to predicting interactions between specific classes of proteins and compounds (Mervin et al., 2015). In other words, the robustness and generalization ability of these models have been greatly reduced when facing the prediction of novel interactions between compounds and proteins not used in model training.

With the accumulation of massive biomedical data, deep learning’s automated feature learning capacity and powerful processing ability have recently shown considerable performance benefits in the fields of image recognition, speech recognition, clinical diagnosis, and drug discovery (Leung et al., 2014; LeCun et al., 2015; Ma et al., 2015). More specifically, protein structure prediction, drug properties (ADMET) prediction, and prediction of synergistic drug combinations are only a few of the areas where deep learning has been employed in drug research due to its benefits in multi-tasking learning (Mayr et al., 2018; Feng et al., 2019; Sturm et al., 2020; Jumper et al., 2021). In terms of CPI prediction, a variety of deep learning frameworks have been utilized, such as a multimodal neural network in DeepCPI (Wan et al., 2019), a graph neural network in CPI-GNN (Tsubaki et al., 2019), and a transformer architecture in TransformerCPI (Chen et al., 2020).

While various deep learning-based models have shown great potential in identifying novel CPIs, there are still several limitations in the previous studies. The first problem is that the interpretability of these models was restricted because of their small CPI training datasets, and they were not comprehensively evaluated by the large-scale bioactivity data that are publicly available. To train the prediction models, many studies also used negative CPI data. Still, it is challenging to collect highly reliable negative samples due to the limited size of inactive CPI data. Second, the issue of hidden ligand bias has been identified in several datasets, such as DUD-E and MUV. Due to this, the predictions end up focusing on compound features rather than interaction features, leading to an overestimation of the prediction performance (Chen et al., 2019; Sieg et al., 2019). Furthermore, a variety of studies lack an evaluation of model generalization ability and applicability, and have not revealed a great generalization ability to external datasets or practical applications, especially for novel CPIs in which compounds and targets are outside the training dataset.

Inspired by these limitations, we, herein, developed a deep learning-based model for predicting CPI with high accuracy, generality, and applicability that can be extended to novel proteins or compounds. We first generated an unbiased large-scale benchmark dataset that integrated multi-source bioactivity data containing a large amount of inactive data. Next, we compared the prediction performance of our deep learning approach with several machine learning algorithms, including K nearest neighbor (KNN), random forest (RF) and extreme gradient boosting (XGBoost). To further assess the generalization ability of our CPI prediction model, we measured the similarity between the tested protein and the training protein using several metrics, identifying the most suitable similarity evaluation metric for evaluating model performance. We then constructed multiple external datasets with novel CPI pairs and applied these external datasets to analyze the model’s performance in terms of similarity evaluation metrics. Finally, the applicability of our model was evaluated by examining its effectiveness in discovering synergistic multi-compound combinations from traditional Chinese medicine (TCM) that possessing a variety of active compounds.

## 2 Materials and methods

### 2.1 Data collection and collation

Large-scale bioactivity data were collected from publicly available databases, such as BindingDB (Gilson et al., 2016), ChEMBL (Mendez et al., 2019), DrugBank (Wishart et al., 2018) and PubChem (Kim et al., 2019). In high-throughput screening, the cut-off for hit detection is typically 10 μM, which was applied to identify active compounds, including highly active and marginally active compounds (Koutsoukas et al., 2013). Therefore, CPIs with half maximal inhibitory concentrations (IC_50_) or inhibition constants (K_i_) ≤ 10 μM in the BindingDB and ChEMBL databases were labeled as positive samples, whereas those pairs with IC_50_ or K_i_ > 10 μM were selected as negative samples. In addition, known interacting drug-target pairs in the DrugBank database were used as positive samples. To ensure accuracy and consistency across different datasets, we employed specific rules during the data collection process. For determining compound activity, a compound was classified as active if it was labeled as active in at least one database. Conversely, if a compound was labeled as inactive across all databases, it was considered inactive. This approach helped minimize the risk of misclassification, especially in cases of variability or uncertainty in experimental results. Next, for compounds with IC_50_ or EC_50_ data available from multiple sources, the smallest value was chosen as the representative IC_50_ or EC_50_ value for that compound. This selection was based on the rationale that a lower IC_50_ or EC_50_ value indicated higher potency, making it the most biologically relevant choice for predicting compound-protein interactions.

The selection of highly reliable negative samples is a challenging task in CPI prediction. The prediction performance of most previous models is negatively impacted by the fact that they have used experimentally unvalidated CPI pairs as negative samples, which can lead to the generation of false negative data. From the BioAssay subdatabase of the PubChem database, more than 294 million bioactivity data with a large number of inactive compound annotations were downloaded and used as negative samples in this study. Subsequently, the collected CPI data were uniformly converted into PubChem CID and UniProt ID identifiers through database mapping files, and duplicate CPI pairs were removed. Compounds with molecular weights less than 100 Da or greater than 1,000 Da were removed to focus on small molecule compounds. Similar compounds acting on the same protein would lead to over-optimistic performance resulting from simple prediction of the model. To reduce hidden ligand bias, Morgan fingerprints of compounds were generated by the RDKit tool. Then, in the compound group with Tanimoto similarity (calculated based on Morgan fingerprints) greater than 0.8 for the same protein, only the compound with the largest number of interactions was retained, while the rest were discarded. Due to the large variation in the number of inactive compounds for different proteins in the PubChem database, only the top 1,000 compounds with a high number of interactions and a Tanimoto similarity of less than 0.8 to other compounds within their compound group were retained as negative samples for the protein.

Furthermore, the collected negative samples were further screened using the compound-target correlation space based interaction prediction model (CTCS-IPM) previously developed by our group (Koutsoukas et al., 2013). Briefly, for the compounds in the negative samples collected above, their known positive targets were gathered. Then, compound descriptors were calculated using Molecular Operating Environment (MOE), and descriptors based on protein amino acid sequences were calculated using PROFEAT. Principal component analysis (PCA) was performed to select feature descriptors. These descriptors were used to construct a compound-target correlation space based on canonical correlation analysis (CCA). Subsequently, for each compound, targets acting on the same compound were defined into a specific target space, and the Euclidean distance between target pairs within the space of compound was calculated. A distance threshold was determined at the upper limit of the 95% confidence interval of average distances among all target pairs within the target space. Then, the Euclidean distance between each protein in the negative sample and the targets within the target space of each compound was calculated. If this distance was greater than the threshold of the target space, it was determined that the given compound does not interact with the protein. The selected negative samples by the CTCS-IPM were then used to construct the prediction model.

### 2.2 Feature representations

#### 2.2.1 Compound feature representations

To extract compound feature representations, SMILES strings of compounds were downloaded from the PubChem database and their 2048 bit Morgan fingerprints with a radius of two were generated via the RDKit tool. In order to avoid the curse of dimensionality caused by excessive features, principal component analysis (PCA) was employed to generate the low-dimensional representations of compound features. The main idea behind PCA is to convert a set of multidimensional variables with certain correlation into some new independent principal components based on linear transformation. This dimensionality reduction method takes into full consideration the correlation between variables while still retaining the majority of the information in the original variable space. According to the cumulative contribution rate, the top 200 principal components were retained as compound feature representations.

#### 2.2.2 Protein feature representations

To extract protein feature representations, Word2vec, a word-embedding technique in Natural Language Processing (NLP) tasks, was used to convert protein sequences into low-dimensional real-valued vectors. Word2vec is a neural network model that learns semantic knowledge from a large number of text corpora in an unsupervised manner and represents the semantic information of words by word vectors after learning the text corpora. To be more specific, a protein sequence was regarded as a document, in which every three non-overlapping amino acid residues was regarded as a word. Following this, low-dimensional embeddings of all possible words were learned by a Continue Bag-of-Words (CBOW) model. For the hyperparameters of the CBOW model, the embedding dimension was set to 100, the number of negative examples was set to 5, the size of context window was set to 5, and the number of training parallelisms was set to 8. Subsequently, the embeddings of all words were fixed, and the representation of protein features was obtained by summing and averaging all word embeddings for the protein.

### 2.3 Model construction

#### 2.3.1 Model architecture

Using Python 3.6 as the platform, deep learning prediction model was constructed using Keras, a TensorFlow-based deep learning framework. The model was developed in sequential mode and used fully connected layers, which consisted of an input layer, hidden layers and an output layer as the basic framework. First, the features of compounds and proteins were combined to create a representation of the features of compound-protein interactions, which were then loaded into the neurons of the input layer. Subsequently, the hidden layers were used to adjust the weights and thresholds to linearly partition the interaction features. The Rectified Linear Unit (ReLU) activation function (Equation 1) in the hidden layers was used to increase the nonlinear relationship between the layers of the neural network and to achieve more efficient gradient descent and back-propagation, which would improve the gradient explosion and vanishing problems. In addition, Batch Normalization layer and Dropout layer were used to accelerate network convergence and control overfitting. Finally, the Sigmoid activation function (Equation 2) in the output layer was applied to generate binary predictions by mapping the predicted values into the range (0, 1).

$$y=\text{Relu}\left(\text{Wx}+b\right)$$ (1)

where *y* is the output value of the hidden layer, *x* is the output value of the input layer or the upper hidden layer, *W* is the weight matrix and *b* is the bias vector.

$$\hat{y}=\text{Sigmoid}\left(W^{\prime }y+b^{\prime }\right)$$ (2)

where $\hat{y}$ is the predicted value of the model, *y* is the activation value of the second hidden layer, $W^{\prime }$ is the weight matrix and $b^{\prime }$ is the bias vector.

The model’s training involved using the compile module to configure the learning process of the model. Specifically, Adam (adaptive moment estimation) was set as the optimizer algorithm, and binary cross-entropy (Equation 3) was set as the loss function.

$$\text{Loss}\left(y_{i},{\hat{y}}_{i}\right)=-\frac{1}{n}\sum_{i=1}^{n}\left[y_{i}\log\left({\hat{y}}_{i}\right)+\left(1‐y_{i}\right)\log\left(1‐{\hat{y}}_{i}\right)\right]$$ (3)

where *n* is the number of samples, $y_{i}$ is the true value of the *i* ^*th*^ sample, and ${\hat{y}}_{i}$ is the predicted value of the *i* ^*th*^ sample.

Hyper parameters are important characteristic data that are usually set artificially for the model. They have a significant impact on model performance. In order to improve the prediction performance of the model, grid search algorithm was applied to seek the optimal hyper parameter combination from the specified hyper parameter spaces, including the number of neurons in the hidden layer, learning rate, dropout rate and epoch number. In particular, the optimization range of the number of neurons in the first hidden layer was {256, 512, 1,024, 2048}, the optimization range of number of neurons in the second hidden layer was {128, 256, 512, 1,024}, the optimization range of learning rate was {0.01, 0.001, 0.0001}, the optimization range of dropout rate was {0, 0.2, 0.5}, and the optimization range of epoch number was {100, 200, 500}. With accuracy as the evaluation metric, 432 models (4*4*3*3*3) constructed with all hyper parameter combinations were evaluated to determine the optimal CPI prediction model.

#### 2.3.2 Model performance metrics

To evaluate the prediction performance of the model, the sensitivity (SEN, Equation 4), specificity (SPE, Equation 5), accuracy (ACC, Equation 6), the area under the Receiver Operating Characteristic curve (AUROC), and the area under the precision-recall curve (AUPR) were used as evaluation metrics. Among them, AUROC and AUPR are widely used to evaluate the performance of binary classifiers against imbalanced datasets. The abscissa of the ROC curve is the false positive rate (FPR, Equation 7) and the ordinate is the true positive rate (TPR, Equation 4). The abscissa of the PR curve is the recall (Equation 4) and the ordinate is the precision (Equation 8).

$$ACC=\frac{TP+TN}{TP+FP+TN+FN}$$ (4)

$$SEN\;/\;TPR\;/\;Recall=\frac{TP}{TP+FN}$$ (5)

$$SPE=\frac{TN}{FP+TN}$$ (6)

$$FPR=\frac{FP}{FP+TN}$$ (7)

$$Precision=\frac{TP}{TP+FP}$$ (8)

where TP is the number of true positive samples, TN is the number of true negative samples, FP is the number of false positive samples, and FN is the number of false negative samples.

### 2.4 Model performance evaluation

#### 2.4.1 Model performance comparison

In the CPI prediction task, we compared our model with three baseline machine learning algorithms, including K nearest neighbor (KNN) model, random forest (RF) model and extreme gradient boosting (XGBoost) model. These prediction methods were all constructed based on scikit-learn library, and trained and evaluated using the same datasets that our deep learning model used. Moreover, the hyper parameters of the other models were also optimized using a grid search algorithm and cross validation. For the KNN model, the hyper parameters of n_neighbors and weights were optimized. For the RF model, the hyper parameters of n_estimators, min_samples_leaf and min_samples_split were optimized. For the XGBoost model, the hyper parameters of max_depth, n_estimators and learning_rate were optimized.

#### 2.4.2 Model generalization ability evaluation by the external validation dataset

The reliability of the prediction results is closely tied to the model’s potential for practical application; however, this has not been done in the case of CPI prediction models, which is a major obstacle to their practical use. Therefore, it is necessary to investigate the prediction performance of our model on novel data (Kaneko and Funatsu, 2014; Kanai et al., 2021). Since the Word2vec model can map semantically similar words to similar word vector spaces (Yang et al., 2018), based on protein feature vectors and amino acid sequences, we measured the similarity between the tested protein and the training protein using Euclidean distance, Cosine similarity and protein sequence identity, and then combined the prediction performance of the model for the tested protein to determine the model’s applicability.

##### 2.4.2.1 Protein similarity evaluation

To clarify the applicability of our model, the influence of protein similarity on its predictive performance was examined. Here, common vector similarity evaluation metrics such as Euclidean distance and Cosine similarity, as well as protein sequence identity, were employed to determine the similarity between modelled proteins. The concept of Similarity Ensemble Approach (SEA) inspired us to develop an efficient method for assessing the similarity between proteins. SEA suggests that two proteins are similar if their active compound sets are similar. To examine whether these metrics could be used to effectively evaluate the protein similarity, we correlated the similarity of protein-protein pair measured by these three metrics with the number of active compounds their shared. Eventually, the most effective vector similarity evaluation metrics was selected for evaluating model performance through an external validation dataset.

##### 2.4.2.2 Model performance evaluation on the external validation dataset

To assess the generalization ability of our model, external CPI data were collected from the BindingDB and ChEMBL databases. As described before, CPI pairs with an IC_50_ or K_i_ ≤ 10 μM were used as positive samples, while CPI pairs with an IC_50_ or K_i_ > 10 μM were used as negative samples. The novel known interacting drug-target pairs in the DrugBank database were used as positive samples. In addition, the KIBA kinase inhibitor bioactivity dataset (Tang et al., 2014) was used as another validation dataset, in which CPI data has been experimentally validated. Then, the binding affinity values were converted to binary values with reference to the recommended KIBA threshold of 12.1 (He et al., 2017). Finally, the analysis of model performance was conducted according to the protein similarity measured by the common vector similarity evaluation metrics.

### 2.5 Experimental validation of our model for discovering synergistic anti-tumor components in TCM

Traditional Chinese medicine (TCM) has attracted considerable attention due to its unique effect on treating diseases. Possessing a variety of active components, TCM function synergistically as a whole to combat diseases. Given the distinctive “multi-compound,” “multi-target,” and “multi-pathway,” characteristics of TCM, unraveling the mechanisms of action of its active compounds remains a challenge. Numerous studies and clinical experiments demonstrated the efficacy of TCM in cancer therapy, which is mainly attributed to the synergistic effects of its multiple ingredients on the complex target networks.

However, incomplete understanding of compound-target interactions has slowed the identification of synergistic combinations derived from TCM. With the use of big data and artificial intelligence technology, we developed a deep learning-based model (DeepMDS), which utilizes the target information of drug combinations and gene expression profiles of cancer cell lines to predict pseudo-IC_50_ values for a chosen cancer cell line (She et al., 2022). To validate our CPI prediction model, the targets of TCM compounds predicted by the CPI model were integrated with known targets and inputted into the DeepMDS to predict synergistic anti-tumor compounds, followed by experimental verification.

Here, we focused on *Astragalus membranaceus* and *Hedyotis diffusaas*, two herbs commonly used in breast cancer treatment. This herb pair was supported by previous clinical research and TCM theory (Liu et al., 2019; Han et al., 2020; Sheik et al., 2021; Zhang et al., 2021). Subsequently, we collected the active compounds of these herbs from the literature and retrieved the targets of these compounds from public databases such as DrugBank Wishart et al. (2018) and PubChem (Kim et al., 2019). Due to the incomplete target data on TCM compounds, we utilized our CPI prediction model to predict additional targets for these compounds, thereby expanding the target dataset. In order to validate the credibility of our model, we first employed the model to predict interactions between the compounds and target proteins. The predicted results were compared with actual interaction relationships to verify their accuracy. Subsequently, this expanded target dataset was used for synergistic anti-tumor compounds prediction. We then generated a variety of multi-compound combinations (ranging from 2 to 5 drugs per combination) using the collected compounds and integrated the target information of the combinations as features inputs into DeepMDS. Additionally, gene expression profiles of the breast cancer cell line MDA-MB-231, which was downloaded from the Gene Expression Omnibus [GEO (Barrett et al., 2013)], were inputted into DeepMDS model.

The DeepMDS model outputted the top-ranked compound combinations, which we then experimentally validated for cell viability in the MDA-MB-231 cell line. We compared the anti-tumor effects of both single and multiple compound combinations at varying concentrations. For multi-compound combinations, the concentration of each compound was equal. The cellular results were compared with the prediction outcome of DeepMDS, and the synergistic effect of each combination was quantified using the Combination Index (CI) calculated via CompuSyn (Chou and Martin, 2007).

## 3 Results

### 3.1 Modeling dataset

The number of CPI data collected from multiple databases as well as the overlap between those databases was shown in Table 1. Following the data preprocessing, a total of 1,014,627 CPI pairs were obtained. This included 346,943 compounds, 1,957 proteins, 485,954 positive samples and 528,673 negative samples. The low-dimensional representations of compound and protein features were matched and integrated to represent the interaction features of CPI pairs. Then, the modelling dataset was randomly split into a training set (80%) and a test set (20%).

**TABLE 1:** The number and overlap of CPI data from multiple databases.

	BindingDB	ChEMBL	DrugBank	PubChem
BindingDB	825,533	558,453	3,158	17,800
	(658,031 + 167,502)			
ChEMBL	67.65%	905,350	3,169	21,478
		(688,649 + 216,701)		
DrugBank	24.40%	24.49%	12,941	0
PubChem	2.16%	2.37%	0	68,259,224

Note: The numbers along the diagonal of the table represented the total number of CPI, pairs collected from each database, the numbers above the diagonal represented the number of overlapping CPI, pairs between databases, and the percentages below the diagonal represented the percentage of overlapping CPI, pairs between databases relative to the smaller database.

### 3.2 Overview of our model

**FIGURE 1:**
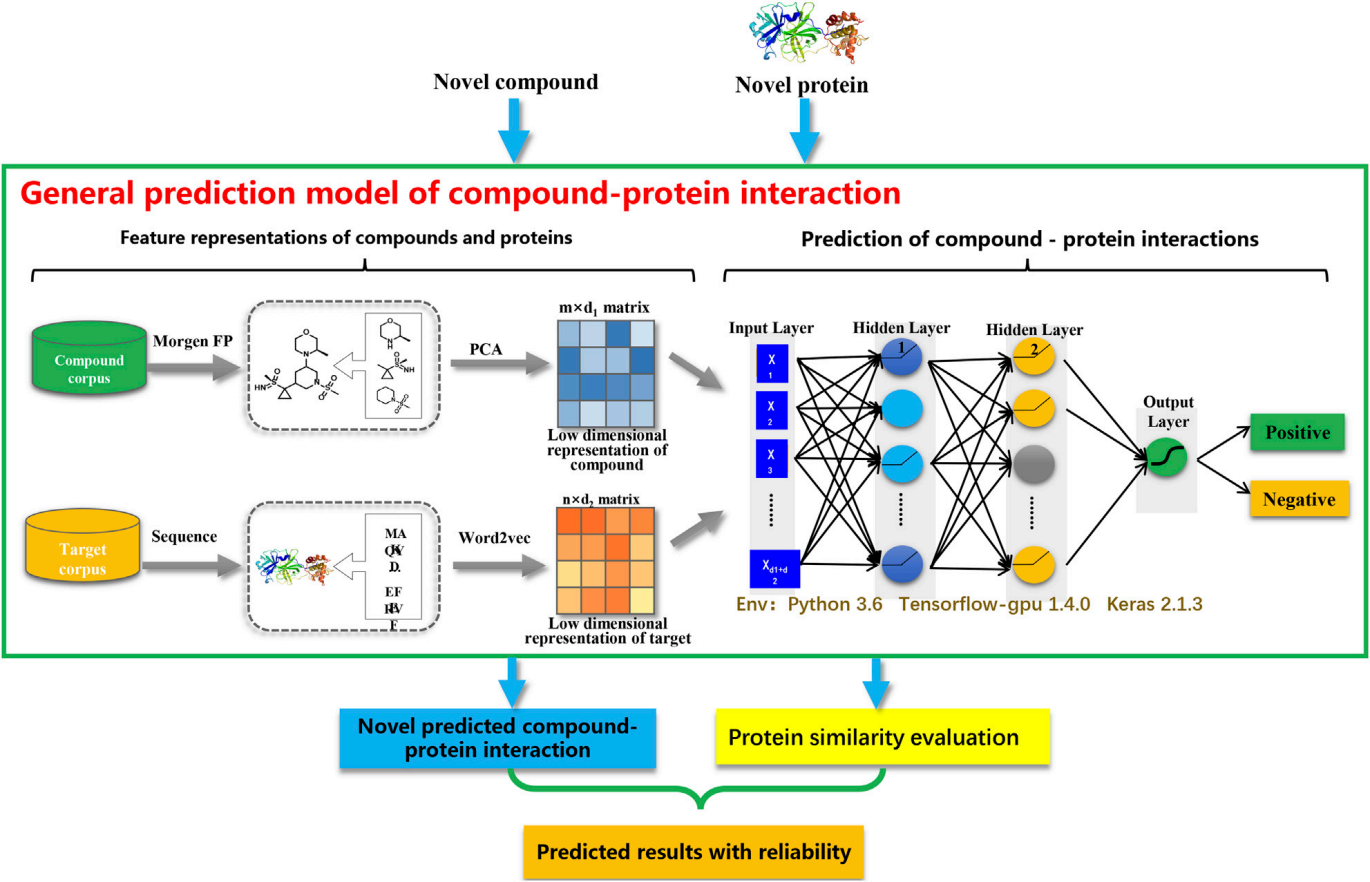
The architecture of the CPI prediction model.

The CPI prediction model mainly comprised two steps: 1) extracting the features of compounds and proteins; and 2) predicting the interactions between compounds and proteins. As illustrated in Figure 1, PCA and Word2vec were applied to automatically extract the low-dimensional representations of compound and protein features, respectively. Subsequently, the representations of compound and protein features were combined to represent the interaction features of CPI pairs. Each CPI pair was represented as an input vector, which consisted of a 200-dimensional representation of compound features and a 100-dimensional representation of protein features. After that, the representation of the interaction features of CPI pairs was fed into a fully connected deep neural network (DNN) to make the predictions.

### 3.3 Model performance

The results from the hyperparameter optimization showed that the best deep learning model had superior prediction performance with an accuracy of 92.74%. The optimal settings for this model were as follows: the number of neurons in the first hidden layer was 2048, the number of neurons in the second hidden layer was 1,024, learning rate was 0.0001, dropout rate was 0.5, and epoch number was 500 (Supplementary Table S1).

We compared the prediction performance of our deep learning model with that of three baseline machine learning models, including KNN, RF and XGBoost. The hyperparameter optimization ranges and the performance evaluation results of these three models were also summarized in Supplementary Table S2, and the five-fold cross-validation accuracies of their optimal models were 90.70%, 89.55% and 91.12%, respectively. Subsequently, we evaluated the prediction performance of our deep learning model and the baseline methods on the same test dataset in terms of five evaluation metrics, including AUROC, AUPR, ACC, SEN and SPE (Table 2). The results showed that our deep learning model outperformed three baseline methods, with an AUROC of 0.98, an AUPR of 0.98, an ACC of 93.31%, an SEN of 93.95%, and an SPE of 92.72%, respectively.

**TABLE 2:** Model performance comparison based on deep learning and three machine learning methods.

	AUROC	AUPR	ACC (%)	SEN (%)	SPE (%)
DNN	0.98	0.98	93.31	93.95	92.72
KNN	0.97	0.96	91.27	91.51	91.04
RF	0.96	0.96	90.08	92.19	88.15
XGBoost	0.97	0.97	91.40	93.50	89.46

### 3.4 Model generalization ability evaluation by the external validation dataset

To further evaluate the accuracy and generalization ability and applicability of the CPI prediction model, external CPI datasets were collected from public databases and the literature. As a result, a BindingDB&ChEMBL dataset of 648,477 novel CPI pairs was constructed from the BindingDB and ChEMBL databases, including 405,385 compounds, 2,601 proteins, 490,243 positive samples and 158,234 negative samples. A Kinase dataset of 240,986 CPI pairs was constructed based on the KIBA kinase inhibitor database, which comprised 51,550 compounds, 467 proteins, 79,018 positive samples and 161,968 negative samples. Another DrugBank dataset containing 2,408 novel CPI pairs was constructed from the DrugBank database, including 1,123 compounds and 1,132 proteins. Subsequently, the similarity between the tested protein and the training protein was determined. This result was combined with the prediction performance of the model for the tested proteins to explore the applicability of the CPI prediction model.

#### 3.4.1 Protein similarity evaluation

**FIGURE 2:**
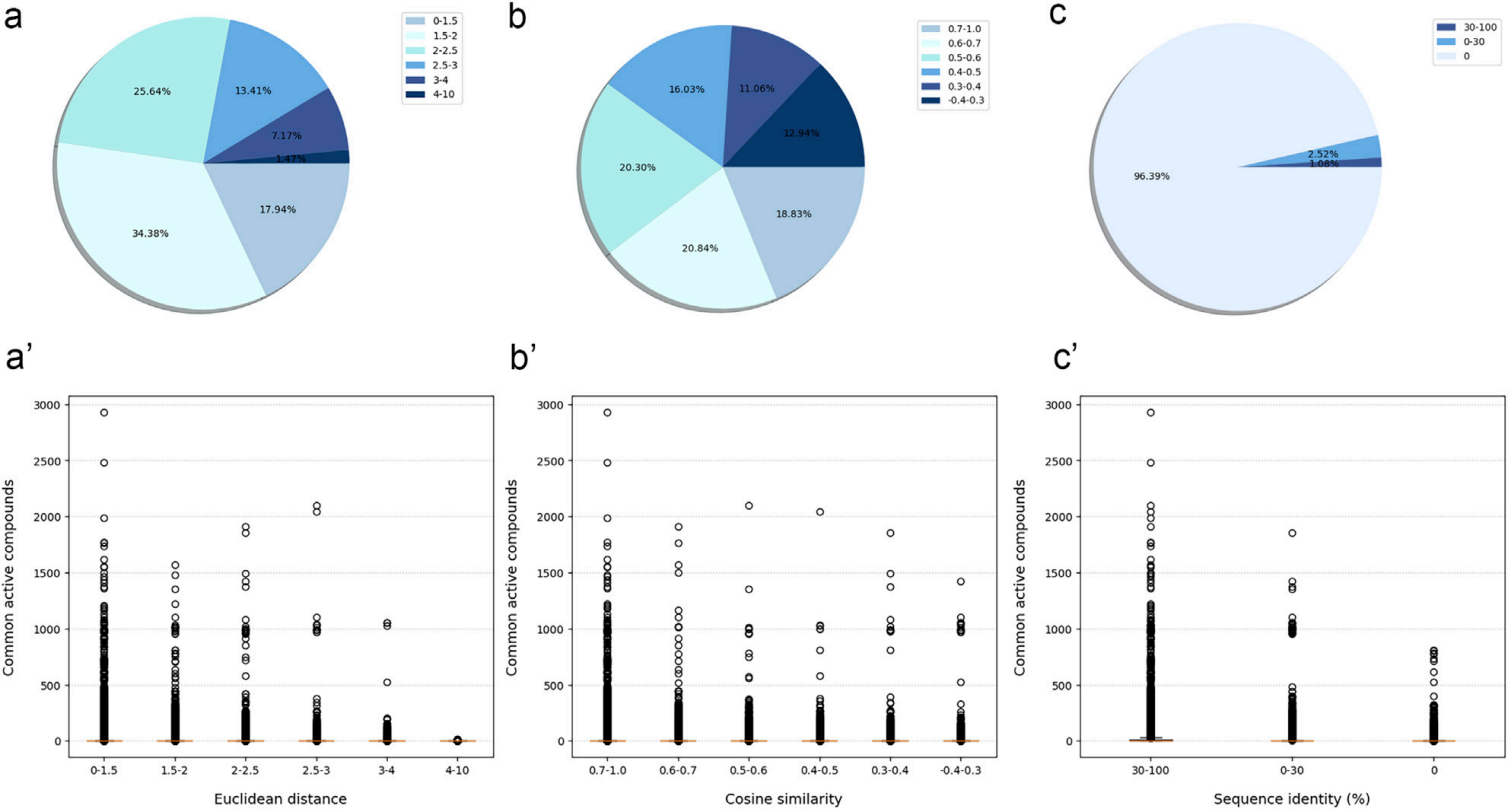
The similarity between modeled proteins was measured by three similarity evaluation metrics, and the relationship with the number of shared active compounds. The similarity and percentage of protein-protein pair measured by Euclidean distance **(A)**, Cosine similarity **(B)** and protein sequence identity **(C)**. The relationship between the similarity of protein-protein pair measured by Euclidean distance **(A')**, Cosine similarity **(B')**, protein sequence identity **(C')** and the number of their shared active compounds. Boxplot represented the interquartile range, with median value as the horizontal orange segment.

Although most protein-protein pairs did not share any active compounds, the evaluation results showed a certain correlation between the number of shared active compounds and the similarity of protein-protein pairs across all similarity evaluation metrics. In details, the more shared active compounds there were, the higher similarity protein-protein pair has (the Euclidean distance approached 0, the Cosine similarity approached 1, and the protein sequence identity approached 100%) (Figure 2).

Compared with the other two metrics, the Euclidean distance might more effectively distinguish between protein-protein pairs with more and fewer shared active compounds. More specifically, the number of shared active compounds was the highest for protein-protein pairs with a Euclidean distance in the interval [0, 1.5]. This number decreased gradually as the Euclidean distance increased, until there were no shared active compounds for protein-protein pairs with the Euclidean distance in the interval [4, 10]. These results suggested that Euclidean distance was a reliable measure of the similarity between proteins. As a result, we used Euclidean distance as the primary metric to measure the similarity between the tested protein and the training protein.

#### 3.4.2 Model performance evaluation on the external validation dataset

**FIGURE 3:**
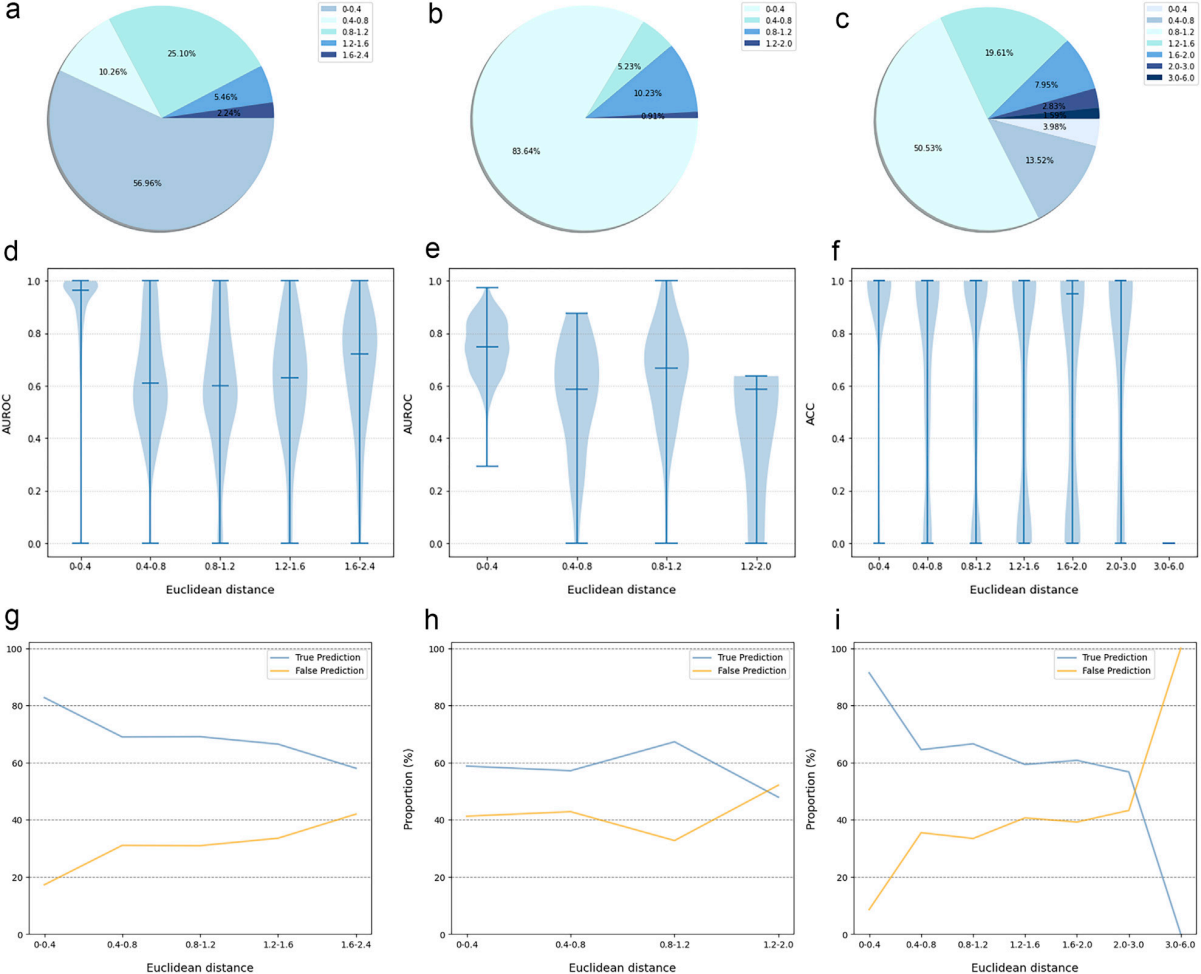
The performance evaluation results of the CPI prediction model on three validation datasets. The percentage of the tested proteins with different Euclidean distance from the training proteins in the BindingDB&ChEMBL dataset **(A)**, the Kinase dataset **(B)** and the DrugBank dataset **(C)**. The relationship between the Euclidean distance of the tested protein from the training protein and the performance of the CPI prediction model on the BindingDB&ChEMBL datasets **(D)**, the Kinase datasets **(E)** and the DrugBank datasets **(F)**. The proportion of CPI pairs with true predictions and false predictions in different Euclidean distance intervals on the BindingDB&ChEMBL datasets **(G)**, the Kinase datasets **(H)** and the DrugBank datasets **(I)**.

**FIGURE 4:**
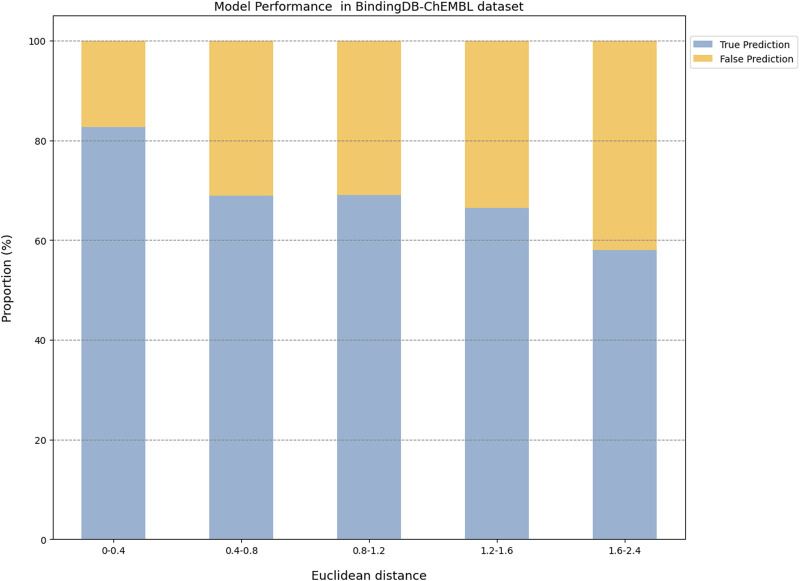
The model performance on the BindingDB and ChEMBL dataset.

The prediction results of the external validation dataset were shown in Figure 3. For the BindingDB&ChEMBL dataset, the CPI prediction model achieved an accuracy of 85.29% and an AUROC of 0.91 for protein whose Euclidean distance from the training protein was in the interval [0, 0.4]. Moreover, the accuracy and AUC for proteins within the intervals of [0.40, 0.67] were (50.45%–62.11%) and (0.60–0.67), respectively (Figure 4).

**FIGURE 5:**
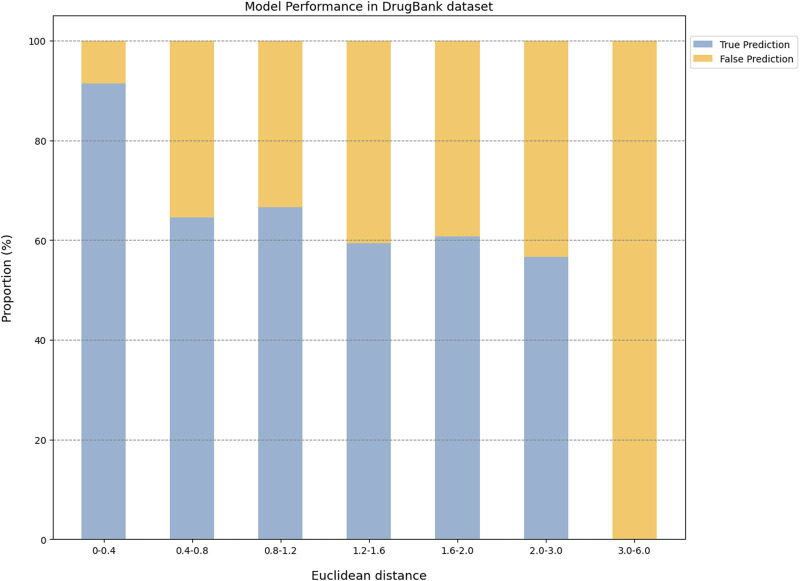
The model performance on the Drugbank dataset.

In addition, the CPI prediction model achieved an accuracy of 92.64% for proteins with Euclidean distances from the modeling protein in the interval [0, 0.4] in the DrugBank dataset. This performance was higher than the accuracy (59.24%–73.13%) of proteins in the interval [0.40, 3.0] (Figure 5).

**FIGURE 6:**
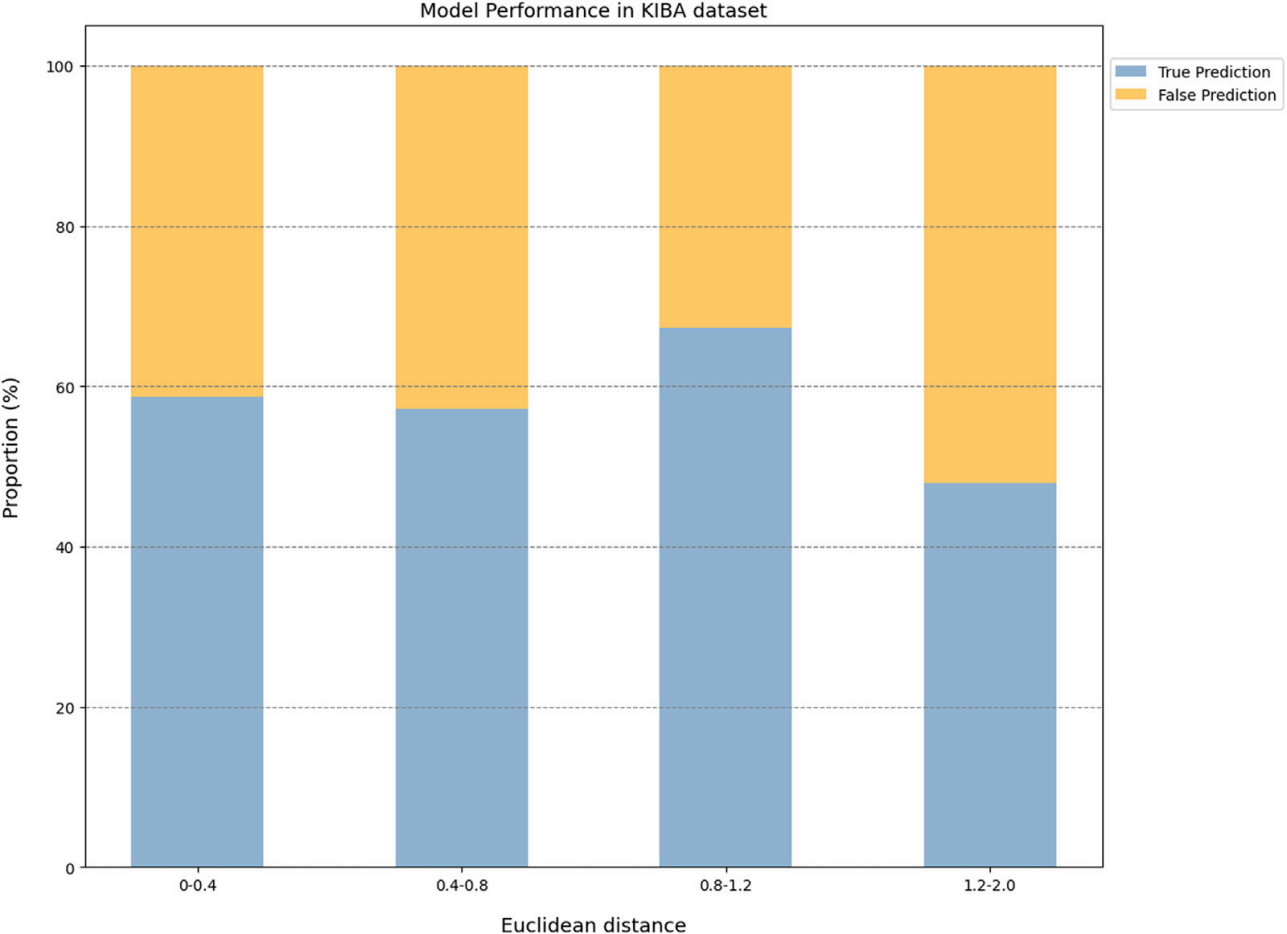
The model performance on the KIBA dataset.

For the KIBA dataset, due to its different mechanism of action from other proteins, the accuracy and the AUC of the CPI prediction model were 64.02% and 0.75, respectively, for proteins with Euclidean distance in [0, 0.40]. Moreover, for the protein with Euclidean distance in [0.40, 2.0], the model reached the accuracy of (26.44%–68.24%) and AUC of (0.45–0.64) (Figure 6).

Taken together, the Euclidean distance between the proteins of most novel CPI pairs and the training proteins was within the interval [0, 0.4], thereby enabling the CPI prediction model to make accurate prediction for these proteins. This finding is evidence of the exceptional generalization capability and applicability of our CPI prediction model. Furthermore, these evaluation results revealed that the sequence similarity between the tested protein and the training protein was correlated with the prediction performance of the CPI prediction model. Specifically, as the sequence similarity increased, the model exhibited an enhanced performance overall, thereby suggesting that the similarity value can serve as a reliable metric for evaluating the prediction results of the model.

### 3.5 Identification of anti-tumor compound combinations from *Astragalus membranaceus* and *Hedyotis diffusaas* herb pair

To demonstrate the application of our CPI model, we attempted to identify potential compound combinations for treating breast cancer from an herb pair of *A. membranaceus* and *H. diffusaas* by combining the CPI model with our DeepMDS model.

We first collected 46 compounds that were identified within *A. membranaceus* and *H. diffusaas* herbs. Then, 558 targets were initially retrieved for 46 compounds from the literature and public databases (Supplementary Table S3). By comparing the predicted compound-protein interaction relationships with the actual ones, our model prediction achieved a recall rate of 87.2%. Using our developed CPI model, we expanded the number of targets in this dataset to 698, generating a more sophisticated compound-target interaction network (Supplementary Table S4). These compounds were then randomly mixed into a library of compound combinations, ranging from 2 to 5 compounds for each combination. Then, the DeepMDS model predicted the candidates from the generated combination library. The predicted pseudo-IC_50_ values of these combinations were summarized in Supplementary Table S5. Among them, two combinations were ranked highest, which exhibited remarkably low pseudo-IC_50_ values (Table 3). The two identified combinations were denoted as Combination I (Comb I) and Combination II (Comb II).

**TABLE 3:** Top 5 predicted drug combinations ranked by Log (pseudo-IC_50_).

No.	Compound-1	Compound-2	Compound-3	Compound-4	Compound-5	Log (pseudo-IC_50_)
1	Epicatechin	Ursolic acid	Quercetin	Aesculetin	Astragaloside IV	−2.313
2	Epicatechin	Ursolic acid	Quercetin	Vanillic acid	Astragaloside IV	−2.200
3	Caffeic acid	Quercetin	Isoquercetin	Glycitein	Astragaloside IV	−1.996
4	Ononin	Quercetin	Aesculetin	Glycitein	Astragaloside IV	−1.974
6	Epicatechin	Asperulosidic acid	Caffeic acid	Quercetin	Rutin	−1.970

To investigate the anti-tumor efficacy of Comb I and Comb II, *in vitro* cellular experiments were carried out using the CCK-8 assay. Both combinations significantly inhibited the proliferation of the human breast cancer cell line MDA-MB-231, showing the low IC_50_ values (Table 4). The results were consistent with the predictions of DeepMDS model. To examine their synergy, we then calculated the CI values of these two combinations. The CI values for Comb I and Comb II were 0.682 and 0.805, respectively, indicating that these two combinations exhibited synergistic anti-tumor effects against MDA-MB-231. Taken together, these results proved that our CPI model could successfully predict the potential interactions between compounds and targets, facilitating the identification of potential inhibitors for a specific therapeutic target.

**TABLE 4:** IC_50_ values of two combinations and individual compound on MDA-MB-231 cells.

Compounds/Multi-compound combinations	IC_50_ (μM)	Compounds/Multi-compound combinations	IC_50_ (μM)
Comb I	19.41	Comb II	23.89
Epicatechin	>1,000	Epicatechin	>1,000
Ursolic acid	6.99	Ursolic acid	6.99
Quercetin	104.60	Quercetin	104.60
Aesculetin	97.11	Vanillic acid	>1,000
Astragaloside IV	>1,000	Astragaloside IV	>1,000

## 4 Discussion

The construction of reliable training datasets is a critical step in CPI prediction; however, the integration of large-scale bioactivity data and the acquisition of reliable negative samples still pose challenges. In this regard, a large number of bioactivity data from various databases, including BindingDB, ChEMBL, DrugBank and PubChem, were integrated to construct a large-scale benchmark dataset that contained selected massive negative samples for CPI prediction. In addition, most CPI prediction models suffer from the issue of the hidden ligand bias, which leads to over-optimistic performance and the prediction performance of the model will be significantly impaired in external validation and practical application scenarios.

To decrease the risk of hidden ligand bias, we removed similar compounds that act on the same protein target to prevent model predictions that rely mainly on compound features rather than interaction features. At the same time, the accuracy and generalization ability of the model were evaluated using multiple novel CPI datasets, and the results demonstrated that our CPI prediction model could effectively learn the interaction features of CPI pairs, rather than the hidden ligand bias in the datasets.

Since a prediction model has a certain scope of application and is not a panacea, it is of great significance to define the applicability potential of the model to maximize its practical utility. In general, since the applicability of the model is closely related to the training dataset, we used the Euclidean distance to evaluate the similarity between the tested protein and the training protein, and we combined this with the prediction performance of the model on the tested protein to explore the prediction space. We found that this model performed best with higher AUROC or ACC for proteins whose Euclidean distance from the training protein was in the interval [0, 0.4]. On the other hand, this model had lower AUROC or ACC for proteins whose Euclidean distance from the training protein was greater than 0.4. Overall, the results suggested that a Euclidean distance of 0.4 could be employed as the critical value for the model applicability potential to measure the reliability of the prediction results.

Our CPI prediction model achieved good prediction performance and generalization ability, even for novel CPI data beyond the prediction space defined by training datasets. Importantly, this capability is not limited by unknown ligand information or protein three-dimensional structures.

Furthermore, our CPI prediction model offer significant advantages over traditional machine learning methods such as KNN, Random Forest, and XGBoost, particularly in handling complex, high-dimensional data and non-linear relationships. These advantages enable our model to predict more accurate CPIs and perform better on unidentified data, making it particularly well-suited for integration into modern drug discovery pipeline.

As an application of our CPI model in TCM, we identified potential synergistic anti-tumor multi-compound combinations from *A. membranaceus* and *H. diffusaas* pair, by using the targets of compounds in the herb pair expanded by the CPI model and subsequently predicted them by DeepMDS. The cellular experimental validation achieved valuable results. The CPI model played a crucial role in this investigation, providing a relatively complete compound-targets interactions. Although there are a number of databases and research reports on drug-target interactions, these resources focused on known drug-target interactions, often neglecting equally important off-target information of compounds. The reason is that off-target information is necessary for understanding the mechanisms of action of compounds and potential side effects. Therefore, we aimed to ensure the completeness and accuracy of compound-target information using the CPI model. These comprehensive data could enable us to precisely identify compound’s mechanism of action. In this study, our CPI prediction model was applied to TCM; as a result, we identified the potential multi-compound combinations that could effectively account for the synergistic therapeutic efficacy of *A. membranaceus* and *H. diffusaas* herb pair. Additionally, efforts will be made to enhance the interpretability of the model by optimizing the model architecture, thus continuously providing new solutions for drug discovery and screening of active compounds in traditional Chinese medicine. To future enhance the applicability and robustness of our CPI prediction model, we will expand our validation efforts beyond breast cancer to include other cancer types such as lung cancer and colorectal cancer. These additional validations will not only confirm the generalization capabilities of our CPI model but also potentially provide novel therapeutic strategies for various cancers.

Interpreting the decision-making process of deep learning models is also an important aspect of their application, especially in fields like drug discovery. Although the primary aim of this study was to develop an accurate model for predicting CPIs, we acknowledge the importance of incorporating interpretability features into deep learning models. Currently, methods such as SHapley Additive exPlanations (SHAP) values, saliency maps, and other visualization techniques are powerful tools that could help explain the underlying mechanisms by which our model arrives at its predictions. These methods can provide valuable insights into which features most affect the outcome of our model, thereby increasing the transparency and trustworthiness of the predictions.

However, the conduct of these interpretability techniques requires additional computational resources and time, which were beyond the scope of the current study. Moreover, the complexity of integrating these methods without compromising the model’s performance requires careful consideration and further study. Therefore, we will explore these interpretability methods in future works to enhance the transparency and utility of our model, making it a more valuable tool in drug discovery.

In conclusion, we constructed a new unbiased large-scale benchmark dataset that contained a large amount of inactive data specific to CPI prediction. With regard to the time- and cost-consuming experimental screening of candidate compounds, we have successfully developed an accurate CPI prediction model that is significantly more effective than other CPI prediction models based on various machine learning algorithms. Furthermore, our model still showed good generalization ability for several external datasets we constructed. Importantly, we defined the applicability potential of our model and enabled the accurate evaluation of the prediction results’ reliability. Our model achieved the best prediction performance for novel CPI data within its prediction space, maximizing its utility in practical applications. Overall, our model has superior prediction accuracy and generalization ability, and is expected to provide technical support for drug discovery, repurposing and the understanding of TCM.

## Data Availability

The code and samples used to support the current study are freely available in the Zenodo repository at the following link: https://doi.org/10.5281/zenodo.8245354.

## Supplementary material

SUPPLEMENTARY TABLE S1 Hyperparameters and evaluation results of the CPI prediction model. This file includes the results of hyperparameter optimization of our prediction model, showing 540 hyperparameter combinations and their evaluation results.

SUPPLEMENTARY TABLE S2 Hyperparameters of three baseline machine learning models. This file includes three sheets that contains the results of hyperparameter optimization for KNN, RF and XGBoost, respectively.

SUPPLEMENTARY TABLE S3 Compounds and Targets collected. This file includes compound and their target derived from database and literature. Compounds and targets are represented by PubChem_CID and Uniprot_ID respectively.

SUPPLEMENTARY TABLE S4 Extended compounds and Targets by the CPI model. This file includes compound and their targets derived from database, literature and CPI prediction. Compounds and targets are represented by PubChem_CID and Uniprot_ID respectively.

SUPPLEMENTARY TABLE S5 Predict outcomes of DeepMDS. This file includes the Top 10000 drug combinations predicted by the DeepMDS.
